# A rare extraction basket impaction: tracing back to biliary tract surgery 10 years ago

**DOI:** 10.1055/a-2697-2470

**Published:** 2025-09-18

**Authors:** Zhengwei Song, Yinda Wang, Shuying Yang, Zhengxiang Zhong, Yiyu Shen, Fei Chen

**Affiliations:** 1 569220 Department of Surgery, The Second Affiliated Hospital of Jiaxing University, Jiaxing, China; 2 569220 Department of Intensive Medicine, The Second Affiliated Hospital of Jiaxing University, Jiaxing, China

A 79-year-old man was hospitalized due to abdominal pain. The patient had a prior history of choledocholithotomy with T-tube drainage, which was performed 10 years earlier, along with three endoscopic retrograde cholangiopancreatography (ERCP) procedures within the past 4 years. Magnetic resonance imaging demonstrated a large stone, approximately 20 × 13 mm, in the distal common bile duct ( Fig. 1 ). After a multidisciplinary discussion, ERCP was selected as the optimal intervention for stone removal.

**Fig. 1:**
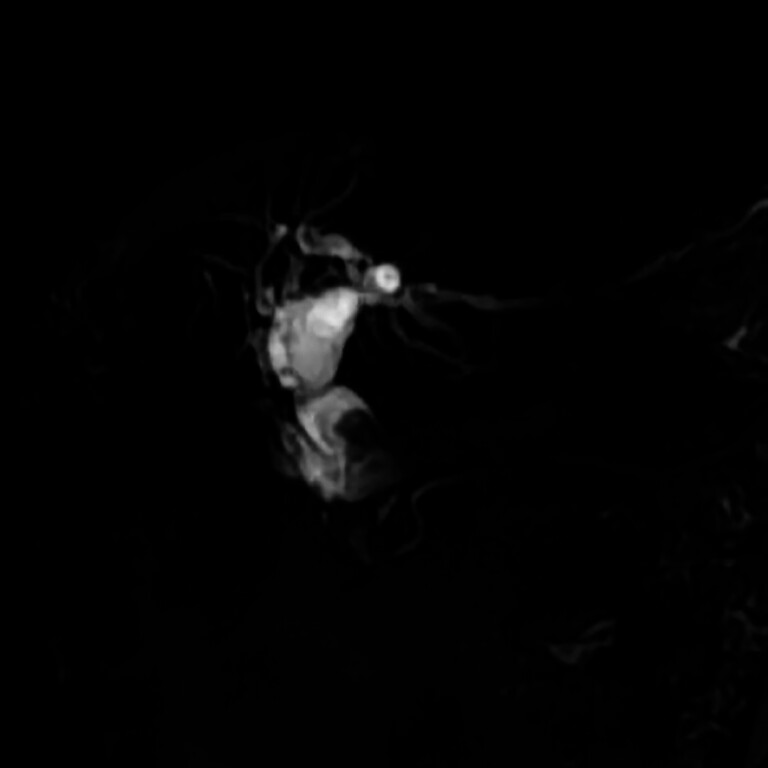
Magnetic resonance imaging showing a large bile duct stone.

Two stones were identified on cholangiogram ( Fig. 2 ). A spiral stone extraction basket (MWB-3X6; Cook Medical, Bloomington, Indiana, USA) was used for the procedure. The initial stone retrieval procedure was successful, extracting a large number of slushy stones ( Fig. 3 ). However, during the final basket cleaning, an unexpected complication occurred: the basket became entangled and incarcerated by a surgical suture ( Fig. 4 **a** ). After extensive efforts, endoscopic scissors (JHY-FG-23-230-A6; Jiuhong Medical, Changzhou, China) were used to disengage the suture near the duodenal papilla ( Fig. 4 **b** , Video 1 ), successfully freeing the incarcerated basket. The entire procedure was completed without adverse events. The patient was discharged on the postoperative day 3, and remained asymptomatic at the 1-month follow-up.

**Fig. 2:**
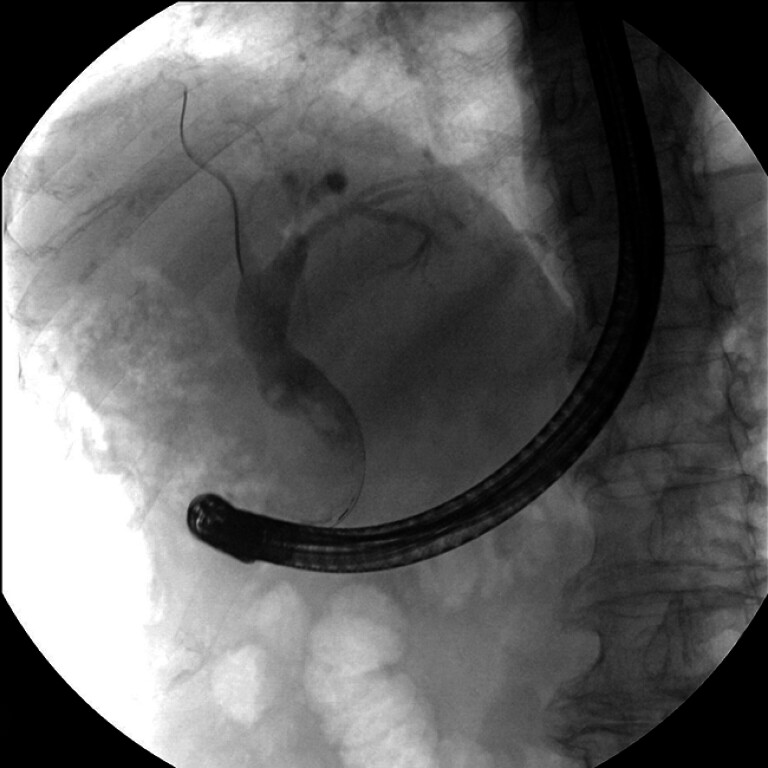
Fluoroscopic image during endoscopic retrograde cholangiopancreatography showing bile duct stones.

**Fig. 3:**
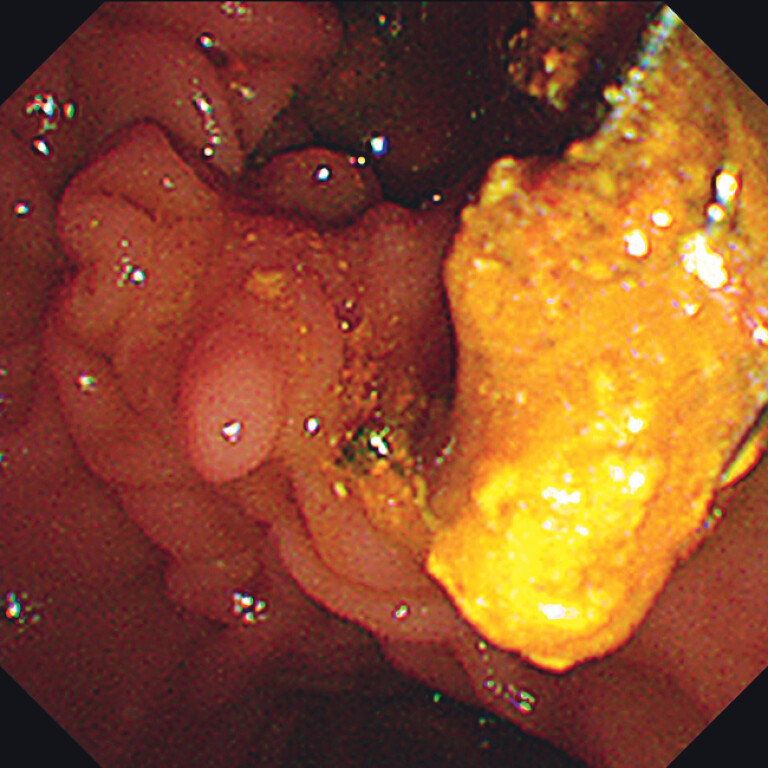
During duodenoscopy, multiple slushy stones were extracted.

**Fig. 4:**
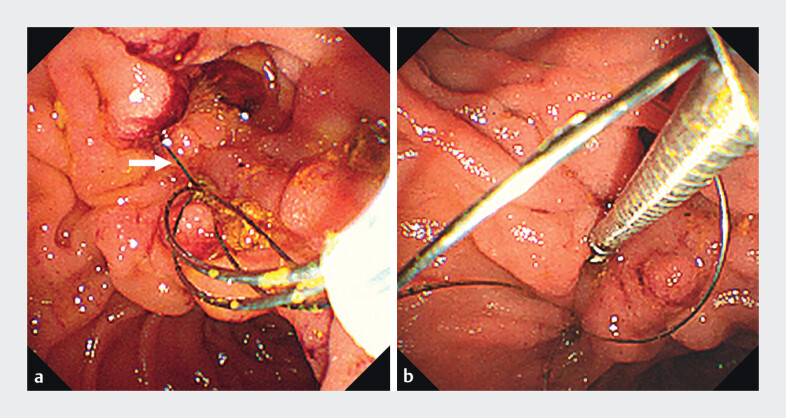
Duodenoscopy images. **a** The basket became entangled and incarcerated by a surgical suture (arrow). **b** The extraction basket was removed using endoscopic scissors.

Use of endoscopic scissors for successful treatment of a rare extraction basket impaction caused by a surgical suture from 10 years earlier.Video 1

This case is presented due to the exceptional rarity of extraction basket impaction caused by a surgical suture from 10 years earlier. Basket incarceration is a rare complication, and almost all previously documented cases are associated with stone entrapment. Prior rescue methods for incarcerated baskets include extracorporeal shock wave lithotripsy 1 , choledochoscopic electrohydraulic lithotripsy 2 , choledochoscopic laser lithotripsy 3 , balloon dilation 4 , and recapturing with another basket 5 . In this elderly patient with a history of biliary surgery, these approaches were clearly unsuitable, and open or laparoscopic surgery would have been excessively invasive. Therefore, we opted for endoscopic scissors, which proved to be an effective treatment for suture-related incarceration.

Endoscopy_UCTN_Code_CPL_1AK_2AF

## Notes

Endoscopy E-Videos https://eref.thieme.de/e-videos
*E-Videos* is an open access online section of the journal *Endoscopy* , reporting on interesting cases and new techniques in gastroenterological endoscopy. All papers include a high-quality video and are published with a Creative Commons CC-BY license. Endoscopy E-Videos qualify for HINARI discounts and waivers and eligibility is automatically checked during the submission process. We grant 100% waivers to articles whose corresponding authors are based in Group A countries and 50% waivers to those who are based in Group B countries as classified by Research4Life (see: https://www.research4life.org/access/eligibility/ ). This section has its own submission website at https://mc.manuscriptcentral.com/e-videos .
